# Elements of Modern Medicines, Etc.

**Published:** 1885-11

**Authors:** 


**Elements of Modern Medicines,** including principles of Pathology and Therapeutics, with many useful memoranda and tables for reference. Designed for the use of students and practitioners. By R. FRENCH STONE, M. D., Professor of Materia Medica and Clinical Medicine in the Central College of Physicians and Surgeons, Indianapolis, etc. New York: D. Appleton & Co., No. 1 Bond street. 1885.

This is a book of 368 pages, bound in "pocket-book" form. We opened it with a prejudice, expecting to find another of the many attempts to condense the science of medicine into a few pages for the benefit of the ignoramus, who, when called to a case, needs a book in his pocket to find out what to do. An examination of the contents shows us that this is a book of a different character. It presents, in a condensed form, the latest accepted views of leading authorities with reference to general pathology and therapeutics, and, although necessarily brief and concise, the author has succeeded in producing a work which may be read by practitioners with interest and profit. Its "handy-book" form will prove convenient for the medical student, and it contains a store of information valuable to them.

